# Europe and the world: boosting international academic cooperation in a time of geopolitical tension and polarization

**DOI:** 10.1093/eurpub/ckab133

**Published:** 2021-08-26

**Authors:** Michaela Vallin, Albin Gaunt, Göran Tomson, Ole Petter Ottersen

**Affiliations:** 1 President’s Office, Karolinska Institutet, Stockholm, Sweden; 2 Department of Medical Biochemistry and Biophysics, Chemical Biology Consortium Sweden, Science for Life Laboratory, Karolinska Institutet, Stockholm, Sweden; 3 International Relations Office, Karolinska Institutet, Stockholm, Sweden; 4 Swedish Institute for Global Health Transformation, SIGHT, Royal Swedish Academy of Sciences, Stockholm, Sweden

The recent European Commission Communication ‘A global approach to research and innovation—Europe’s strategy for international cooperation in a changing world’^1^ acknowledges the unprecedented need to cooperate across borders to strengthen the EU’s long-term research and innovation value chains and meet the sustainable development goal (SDGs) of the United Nations 2030 Agenda. The aim is to achieve an open research and innovation environment based on European rules and values and to ensure a level-playing field in international cooperation. To reach these aims, the EU intends to build an open strategic autonomy, develop its science diplomacy and use intellectual property (IP) in a smart way.

The gist of the Communication is captured by the following sentence: ‘The EU should more assertively promote a level playing field and reciprocity to respect fundamental values and principles, to protect the use of IP rights, to ensure the security of supply, and to encourage fair innovation ecosystems not distorted by undue rules or foreign subsidies […]’ The increased ‘assertiveness’ on the part of EU mirrors the move towards self-reliance and self-sufficiency in other parts of the world.^2^ How will international academic cooperation fare in a world that is becoming increasingly polarized, with a palpable risk of politicization of science? What can EU do to balance the need for global exchange of knowledge and ideas with the perceived need for increased security and autonomy?

## Global academic collaboration must be based on global values

The most disappointing aspect of the Communication is the reference to ‘European values’. In a geopolitically turbulent and increasingly polarized world it is understandable that governments grow introspective when it comes to core values. However, we should cling to the idea that values are global and generic. Insisting on values that are inherently ‘European’ is tantamount to taking the high moral ground and is incongruous with the ‘level playing field’ that the Communication asks for. With a ‘global approach’ to cooperation the EU should refer to the Human Right Convention, the Magna Charta Universitatum and other covenants of global commitment. The least we need is a cold war on values. We should insist on the idea that values are global, as is the quest for new understanding and insight.

## Building capacity requires IP flexibility

International cooperation and tech transfer rely on IP rules and regulations. The Communication signals that EU will assertively ‘protect the use of IP rights’ and develop a code of practice on ‘smart use of IP’. The text offers no clue as to the substance of the latter term. There are reasons to be vigilant: the EU contested the proposed waiver from certain provisions of the World Trade Organization’s Agreement on Trade-Related Aspects of IP Rights.^3^ This proposal was put forward in October last year and aimed to facilitate production of vaccines in poor countries. It is not easy to reconcile EU’s stance on this proposal with the statement in the Communication that ‘the Commission will enhance its commitments to strengthen health systems, global health security, increase access to medicines and health products, notably though research, innovation, capacity building and support to local production’. Africa still imports 99% of its vaccines due to limited production capacity. Considering this challenge ‘smart use of IP’ should translate into ‘flexible use of IP’ to help reduce the blatant technology gap that now exists between rich and poor nations and help open for academic cooperation on capacity building and tech transfer. This would be an important step towards the realization of the SDGs and an important commitment to the fundamental ethical values that figure so prominently in the Communication.

## Security of supply vs. international academic collaboration: we need to retain global ‘knowledge supply chains’

The Communication states that EU should more assertively ‘ensure the security of supply’, referring to the perceived need to be more self-reliant and less dependent on commercial supply chains in politically unstable times.^4^ In many ways, this is understandable—more diversified supply chains would amount to an increased resilience in a time of crisis. We know however that along commercial supply chains there is a flux of academic exchanges—what we here describe as parallel ‘knowledge supply chains’. Thus when looking ahead there is an obvious risk that severing commercial supply chains will also interrupt adjoining knowledge supply chains—causing a ‘destructive interference’ between the two.

We recommend that the political follow-up on this Communication be coupled to an awareness of the interdependence between trade, supply chains and scientific exchange. More than ever do we need open channels for scientific exchange between EU and the rest of the world. COVID-19 has been a real eye-opener: key to scientific development is the willingness to share data, reagents and know-how across borders. There has been a shift in attitude from being the first to publish to being the first to share. We should embrace this development and avoid policies that inadvertently impact global academic cooperation.

Open knowledge supply channels are a prerequisite for what stands as an overriding goal for EU policies in general and highlighted specifically in the Communication: strengthened innovation. A recent paper in The Economist states that Europe is lagging behind USA and Asia as a hub for transnational companies.^5^ Boosting innovation requires free exchange of ideas between EU and the rest of the world. An EU with less than 6% of the world’s population is much dependent on the influx of knowledge and ideas from the world at large.

## The need for responsible internationalization

We must not be naive. Ideas do flow along academic cooperative links, but so do security issues. The secondary use of knowledge for military purposes is a case in point and AI research in the realms of health and other disciplines raises ethical questions that cannot be ignored. These challenges place a heavy responsibility on academic institutions. There is an increasing need for insight in ethical and security implications of international academic collaboration. This must be developed within the academic institutions themselves to safeguard institutional autonomy and pre-empt interference from national or supranational authorities. There should be a focus on ‘responsible internationalization’ with due attention to ethics and academic freedom and with support of expertise that can help assess ethical and security challenges *ex ante—*before a memorandum of understanding is signed or an academic collaboration is formalized.

## Conclusion

It is excellent that the EU commission has put global cooperation on the agenda at a time when global cooperation in the realm of health and other fields is threatened by increased protectionism and distrust. The current Communication signals high ambitions when it comes to future academic collaborations. However, ambiguous terms abound: open strategic autonomy, reciprocal openness, level playing field, rules based cooperation—just to name a few. On the positive side the Communication leaves many issues open for discussion by the member states. Everybody would gain from an open an honest discussion of conflicting goals and from an explicit definition of the key terms that constitute the reference frame of the current debate. The COVID-19 pandemic has showed how dependent we are on global exchange of ideas and knowledge. The challenge is to ensure that any measures taken to safeguard security and autonomy do not detract from the need to maintain and expand international academic collaboration. Academia must be vigilant in this respect. We must engage in the seminal discussion on how to foster and promote academic cooperation in a turbulent and polarized world and insist on transparency. It is essential that academic channels be kept open, even when commercial and diplomatic channels are subjected to geopolitical stress.

## Funding

None.

*Conflicts of interest:* O.P.O. has contributed to EUA’s input to the Communication.^1^

## References

[1] European Commission. *Communication on the Global Approach to Research and Innovation*. Brussels: European Commission, 2021. Available at: https://ec.europa.eu/info/sites/default/files/research_and_innovation/strategy_on_research_and_innovation/documents/ec_rtd_com2021-252.pdf (15 July 2021, date last accessed).

[2] A confident China seeks to insulate itself from the world. The Economist, March 2021. Available at: https://www.economist.com/china/2021/03/11/a-confident-china-seeks-to-insulate-itself-from-the-world (12 July 2021, date last accessed).

[3] Ekström AM, Berggren C, Tomson G, et al The battle for COVID-19 vaccines highlights the need for a new global governance mechanism. Nat Med 2021;27:739–40. 33707776 10.1038/s41591-021-01288-8

[4] European Commission. Trade Policy Review – An Open, Sustainable and Assertive Trade Policy. Brussels: European Commission, 2021. Available at: https://trade.ec.europa.eu/doclib/docs/2021/february/tradoc_159438.pdf (15 July 2021, date last accessed).

[5] Europe is now a corporate also-ran. Can it recover its footing? The Economist, June 2021. Available at: https://www.economist.com/briefing/2021/06/05/once-a-corporate-heavyweight-europe-is-now-an-also-ran-can-it-recover-its-footing (8 July 2021, date last accessed).

